# A potent weighted risk model for evaluating the occurrence and severity of diabetic foot ulcers

**DOI:** 10.1186/s13098-021-00711-x

**Published:** 2021-08-31

**Authors:** Lu Shi, Huiyi Wei, Tianxiao Zhang, Zhiying Li, Xiaoxian Chi, Dandan Liu, Dandan Chang, Yueying Zhang, Xiaodan Wang, Qingbin Zhao

**Affiliations:** 1 Department of Geratology, The First Affiliated Hospital of Xi’an Jiaotong University, Xi’an, 710061 Shaanxi China; 2 Yan’an University, Yan’an, 716000 Shaanxi China; 3 Department of Epidemiology and Biostatistics, School of Public Health, Xi’an Jiaotong University Health Science Center, Xi’an, 710061 Shaanxi China; 4 Department of Geratology, Ninth Hospital of Xi’an, Xi’an, 710061 Shaanxi China; 5 Department of Endocrinology, The First Affiliated Hospital of Xi’an Medical College, Xi’an, 710077 Shaanxi China

**Keywords:** Risk model, Diabetic foot ulcers, Risk factor, Random forest

## Abstract

**Background:**

Diabetic foot ulcer (DFU) is a serious chronic complication of diabetes. This study aimed to establish weighted risk models for determining DFU occurrence and severity in diabetic patients.

**Methods:**

This was a multi-center hospital-based cross-sectional study. A total of 1488 diabetic patients with or without an ulcer from three tertiary hospitals were included in the study. Random forest method was used to develop weighted risk models for assessing DFU risk and severity. Receiver operating characteristic curves were used to validate the models and calculate the optimal cut-off values of the important risk factors.

**Results:**

We developed potent weighted risk models for evaluating DFU occurrence and severity. The top eight important risk factors for DFU onset were plasma fibrinogen, neutrophil percentage and hemoglobin levels in whole blood, stroke, estimated glomerular filtration rate, age, duration of diabetes, and serum albumin levels. The top 10 important risk factors for DFU severity were serum albumin, neutrophil percentage and hemoglobin levels in whole blood, plasma fibrinogen, hemoglobin A1c, estimated glomerular filtration rate, hypertension, serum uric acid, diabetic retinopathy, and sex. Furthermore, the area under curve values in the models using plasma fibrinogen as a single risk factor for determining DFU risk and severity were 0.86 (sensitivity 0.74, specificity 0.87) and 0.73 (sensitivity 0.76, specificity 0.58), respectively. The optimal cut-off values of plasma fibrinogen for determining DFU risk and severity were 3.88 g/L and 4.74 g/L, respectively.

**Conclusions:**

We have established potent weighted risk models for DFU onset and severity, based on which precise prevention strategies can be formulated. Modification of important risk factors may help reduce the incidence and progression of DFUs in diabetic patients.

**Supplementary Information:**

The online version contains supplementary material available at 10.1186/s13098-021-00711-x.

## Background

Diabetic foot ulcer (DFU) is a severe diabetic complication that consists of lesions in the deep tissues associated with neurological disorders and peripheral arterial disease (PAD) in the lower limbs [1]. The global prevalence of diabetic foot disease is 6.3%, and its prevalence in China is about 4.1% [2]. A recent study reported that the 1-, 2-, and 5-year survival rates of diabetic foot disease were 81%, 69%, and 29%, respectively, and the association between mortality and DFUs was stronger than that between mortality and any other macrovascular disease [3]. Moreover, up to one-third of diabetes expenditure is known to be on lower-limb-related problems in the US [4].

The pathogenesis of DFUs is complex. Diabetes has been commonly associated with a series of micro- and macro-vascular changes that manifest as a wide range of complications. DFUs are a devastating component of diabetes progression, and an estimated 15% of diabetic patients develop foot ulcers during the course of their disease [5, 6]. The commonly identified risk factors predisposing to the development of foot ulcers include poor glycemic control, peripheral neuropathy, and PAD. The biochemical basis of ulceration is caused by a combination of components that together lead to tissue breakdown. Patients with DFUs are older, have a lower body mass index (BMI), longer diabetic duration, are hypertensive, have diabetic retinopathy (DR), and a history of smoking when compared to patients without ulcers [2]. Moreover, foot ulcer severity has been associated with the worst outcomes and a higher rate of lower limb amputation among diabetic patients. Therefore, preventing the occurrence and progression of foot ulcers is very important to reduce poor outcomes in diabetic patients.

Although the International Working Group on the Diabetic Foot has devised a risk stratification system that can be used to assess the risk of developing foot ulcers in diabetic patients [7], which was mainly based on foot pathological characteristics, the guidelines did not take into account systemic features of diabetic patients, which may predate the presentation of foot ulcers. Furthermore, previous studies in this area have only focused on a single risk factor for DFU onset or severity; there is little research on the weighted relationship among various risk factors related to DFUs. It is not clear what risk factors are important in assessing the occurrence and progression of DFUs among diabetic patients. Studies are urgently required to better define the categories of patients that will benefit from preventative interventions and the specific types of interventions to be prioritized. The objective of this study was therefore to establish weighted risk models for determining DFU onset and progression, and to calculate the optimal cut-off values for important risk factors. Our findings will benefit the development of strategies for the prevention and treatment of DFUs, and reduce poor outcomes in DFU patients.

## Methods

### Study design

This study was a multi-center cross-sectional study. In this study, all consecutive patients with DFUs from January 2013 to December 2020 and diabetic patients without foot ulcers randomly selected during the same period from three tertiary general hospitals were included as the study subjects. The three hospitals were the First Affiliated Hospital of Xi’an Jiaotong University, the First Affiliated Hospital of Xi’an Medical College, and the Ninth Hospital of Xi’an. The methodology for blood biochemical tests was consistent across the three hospitals. This study was approved by the Ethics Committees of the three hospitals and was performed according to the Declaration of Helsinki. Written informed consent was obtained from each participant.

### Study population

A total of 1488 participants from three hospitals were included in the study. Adult patients diagnosed with diabetes and foot ulcers were eligible for participation, and these patients were not treated with antibiotics prior to admission. The exclusion criteria for patients with DFUs included (1) patients with other systemic or localized infectious diseases, (2) patients with hematological diseases, (3) patients with systemic inflammatory diseases, (4) patients with rheumatic immune diseases, and (5) patients receiving ongoing immunosuppressive treatment. Finally, 725 consecutive patients with DFUs comprised the case group. Since all DFU patients had type 2 diabetes, we randomly selected 763 patients with type 2 diabetes without foot ulcers who were hospitalized during the same period as the control group. The diagnosis of diabetes was based on the World Health Organization criteria, which stated: fasting plasma glucose, ≥ 126 mg/dL (7.0 mmol/L) or oral glucose tolerance test 2-h plasma glucose, ≥ 200 mg/dL (11.1 mmol/L). In addition, type 1 diabetes, gestational diabetes, and other specific types of diabetes were excluded in both groups. DFUs were defined as diabetic patients who developed wounds secondary to neuropathy with or without biomechanical abnormalities, PAD, or both [8]. The Wagner classification system was used to evaluate the severity of DFUs [9]; patients with a Wagner grade < 3 were defined as mild DFUs, while those with a Wagner grade ≥ 3 were defined as severe DFUs.

### Candidate risk factors

A total of 17 variables were selected based on the previous literature and expert opinions as the candidate risk factors of DFU onset and severity (Additional file 1: Table S1). All 17 variables were classified into three categories. Demographic data of the study subjects comprised five variables including sex, age, BMI, smoking status, and family history of diabetes. Clinical information comprised another five variables including duration of diabetes; history of coronary heart disease, stroke, and hypertension; and DR status. In addition, we also included seven biochemical indexes which may contribute to the risk assessment of DFU onset and severity. These biochemical indexes included whole blood level of hemoglobin, neutrophil percentage, hemoglobin A1c (HbA1c), serum level of albumin and uric acid (SUA), plasma fibrinogen level, and estimated glomerular filtration rate (eGFR).

BMI was calculated as weight (kg)/height (m^2^), which was measured at admission. Hypertension was defined as systolic/diastolic blood pressure ≥ 140/90 mmHg or the use of any antihypertensive medication. History of coronary heart disease was confirmed by medical records or defined by the history of angina pectoris or myocardial infarction, any positive cardiac stress test result, or pathological signs on coronary angiography [10]. History of stroke was defined as the presence of any neurologic deficiency event with or without sequelae [10]. Binocular fundus examinations were performed using wide-area fundus photography, optical coherence tomography, and fundus fluorescein angiography in each participant. The images were analyzed by ophthalmologists and DR was graded and staged as follows: (1) no apparent DR; (2) mild non-proliferative DR (NPDR), meaning microaneurysms alone; (3) moderate NPDR, meaning more than microaneurysms but less than severe NPDR; (4) severe NPDR, indicated by intraretinal hemorrhage in each of the 4 quadrants, venous beading in ≥ 2 quadrants, or intraretinal microvascular abnormalities in ≥ 1 quadrant, but no proliferative DR (PDR); and (5) evidence of PDR, indicated by neovascularization and/or vitreous preretinal hemorrhage [11].

Venous blood was collected from all participants after an overnight fast of at least 10 h for biochemical tests. SUA, serum creatinine, and albumin levels were determined using automatic biological analyzer (HITACHI, LABOSPECT008). The percentage of neutrophils and hemoglobin in whole blood were measured using automatic analyzer (MINDRAY 6800 plus). HbA1c was determined using high performance liquid chromatography [12]. Plasma fibrinogen was determined by Class coagulation method [13]. eGFR was estimated with the Chronic Kidney Disease-Epidemiology Collaboration equation [14].

### Statistical analysis

**Fig. 1:**
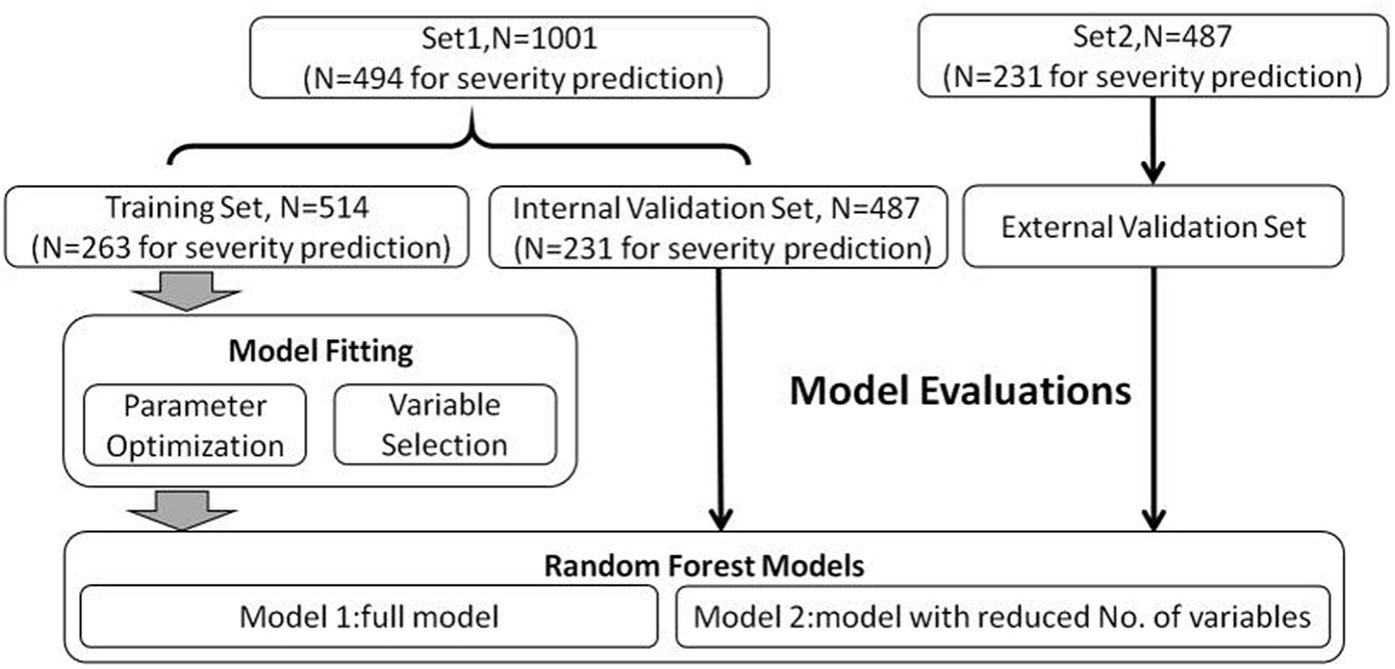
Flow chart for statistical analysis

In the present study, our sample size was mainly referred to two types of previous reports: (1) a methodological study using simulation techniques [15] and (2) a similar study previously conducted [16]. Two independent datasets were collected for data analysis (Set1 and Set2) in this study. Set1 was from the patient data of the First Affiliated Hospital of Xi’an Jiaotong University, while Set2 was from the patient data of the two other hospitals. Set1 was randomly divided as the training (N = 514) and internal validation set (N = 487) to fit machine learning models and optimize the relevant parameters for the evaluation of DFU risk. While Set2 was used as an external validation set (N = 487) to evaluate the model performance. Similar analysis strategy was applied for the assessment of DFU severity. The analysis flow chart is shown in Fig. 1.

A decision tree is a flowchart-like structure. It is used for solving both classification and regression problems in the form of trees that can be incrementally updated by splitting the dataset into smaller datasets. A random forest (RF) is a supervised learning algorithm which is built by multiple decision trees. Stable and accurate evaluation results could be produced by merging all these decision trees together. In the present study, we have constructed RF models to evaluate the risk and severity of DFUs. All the 17 variables were included in the RF model. The performance of RF models was evaluated by receiver operating characteristic curves. Indicators including area under curve (AUC), sensitivity, specificity, and accordance rate were calculated. The 95% confidence intervals (CI) for these indicators were obtained by 1000 bootstrapping. To evaluate the potential effects of overfitting, we have examined RF models with reduced number of variables. RF models with 8 variables (for the assessment of DFU risk) and 10 variables (for the assessment of DFU severity) were constructed. In addition, to investigate the clinical significance of the plasma level of fibrinogen for determining DFU risk and severity, we also evaluated its performance in a single variable model using the combined datasets, Set1 and Set2.

Continuous variables were presented as mean ± standard deviation (SD), and differences between the two groups were compared using a t test. Categorical variables were presented as percentages, and differences between the two groups were compared using the Chi-squared test. A P value of < 0.05 was considered statistically significant. R project and relevant packages (version 3.6.3) were utilized for data analysis. Additional detailed information concerning parameter optimization and model fitting is summarized in Additional file 1: Texts.

## Results

### Demographic and clinical information

The demographic and clinical information of our study subjects is summarized in Table 1. Of the 17 variables included in the RF model, 16 variables showed significant differences between controls and DFU cases in Set1. On the other hand, only 12 variables showed significant differences between controls and DFU cases in Set2. Stroke, hypertension, DR, family history of diabetes, and SUA levels were only identified to be significantly different between controls and cases in Set1. While sex was found to be unevenly distributed between controls and DFU cases in Set2 alone. These results indicated that the two datasets had systematic differences.

**Table 1:** Demographic and clinical characteristics of the study subjects

Variables	Set1		Statistics	*P*-value	Set2		Statistics	*P*-value
	Cases (N = 494)	Controls (N = 507)			Cases (N = 231)	Controls (N = 256)		
Sex (%)								
Male	358 (72)	358 (71)			179 (77)	170 (66)		
Female	136 (28)	149 (29)	χ^2^ = 0.3	0.56	52 (23)	86 (34)	χ^2^ = 6.8	0.009
Age, years	61.7 ± 12.0	55.8 ± 11.2	*t* = 8.0	3.42 × 10^–15^	64.0 ± 12.3	59.7 ± 12.1	*t* = 3.8	1.00 × 10^–4^
BMI, kg/m^2^	23.4 ± 3.4	24.7 ± 3.4	*t* = − 5.9	6.34 × 10^–9^	23.4 ± 3.7	24.6 ± 3.2	*t* = − 4.0	8.68 × 10^–5^
Diabetes duration, years	12.5 ± 7.9	9.2 ± 7.2	*t* = 7.0	5.60 × 10^–12^	11.4 ± 8.0	7.7 ± 6.9	*t* = 5.5	8.15 × 10^–8^
Coronary heart disease (%)								
Yes	99 (20)	68 (13)			73 (32)	53 (21)		
No	395 (80)	439 (87)	χ^2^ = 7.4	0.006	158 (68)	203 (79)	χ^2^ = 7.0	0.008
Stroke (%)								
Yes	95 (19)	19 (4)			46 (20)	43 (17)		
No	399 (81)	488 (96)	χ^2^ = 57.9	2.74 × 10^–14^	185 (80)	213 (83)	χ^2^ = 0.6	0.441
Hypertension (%)								
Yes	272 (55)	213 (42)			118 (51)	122 (48)		
No	222 (45)	294 (58)	χ^2^ = 16.5	4.77 × 10^–5^	113 (49)	134 (52)	χ^2^ = 0.4	0.506
Diabetic retinopathy (%)								
Yes	282 (57)	197 (39)			58 (25)	53 (21)		
No	212 (43)	310 (61)	χ^2^ = 32.6	1.14 × 10^–8^	173 (75)	203 (79)	χ^2^ = 1.1	0.294
Family history of diabetes (%)								
Yes	175 (35)	228 (45)			43 (19)	49 (19)		
No	319 (65)	279 (55)	χ^2^ = 9.1	0.003	188 (81)	207 (81)	χ^2^ = 0.001	0.974
Smoking status (%)								
Yes	225 (46)	269 (53)			111 (48)	98 (38)		
No	189 (54)	318 (47)	χ^2^ = 6.7	0.01	120 (52)	158 (62)	χ^2^ = 4.3	0.037
Hemoglobin, g/L	117.3 ± 22.5	136.8 ± 18.5	*t* = − 15.0	< 2.2 × 10^–16^	124.7 ± 20.5	138.2 ± 18.3	*t* = − 7.6	1.27 × 10^–13^
Neutrophil percentage, %	72.7 ± 11.7	60.6 ± 9.2	*t* = 18.3	< 2.2 × 10^–16^	69.1 ± 10.9	60.2 ± 10.4	*t* = 9.3	< 2.2 × 10^–16^
Serum albumin, g/L	33.6 ± 6.2	40.1 ± 4.3	*t* = − 19.3	< 2.2 × 10^–16^	35.3 ± 5.5	41.3 ± 8.1	*t* = − 9.7	< 2.2 × 10^–16^
Serum uric acid, μmol/L	306.1 ± 108.8	316.4 ± 81.1	*t* = − 1.7	< 2.2 × 10^–16^	316.0 ± 102.4	320.2 ± 94.2	*t* = − 0.5	0.637
Plasma fibrinogen, g/L	5.3 ± 1.7	3.0 ± 0.8	*t* = 27.2	< 2.2 × 10^–16^	4.4 ± 1.5	3.2 ± 0.9	*t* = 10.9	< 2.2 × 10^–16^
eGFR, mL/min/1.73m^2^	91.9 ± 40.2	106.2 ± 27.9	*t* = − 6.5	1.06 × 10^–10^	91.2 ± 35.1	112.1 ± 38.9	*t* = − 6.2	9.71 × 10^–10^
HbA1c, %	9.1 ± 2.3	8.4 ± 2.0	*t* = 5.3	1.12 × 10^–7^	9.6 ± 2.6	8.9 ± 2.3	*t* = 3.2	1.56 × 10^–3^
Clinical severity (%)								
Severe	253 (51)	–			88 (38)	–		
Mild	241 (49)	–	–	–	143 (62)	–	–	–

Continuous variables were presented as mean ± SD

*HbA1c* Hemoglobin A1c

### RF models and performance evaluation

**Fig. 2:**
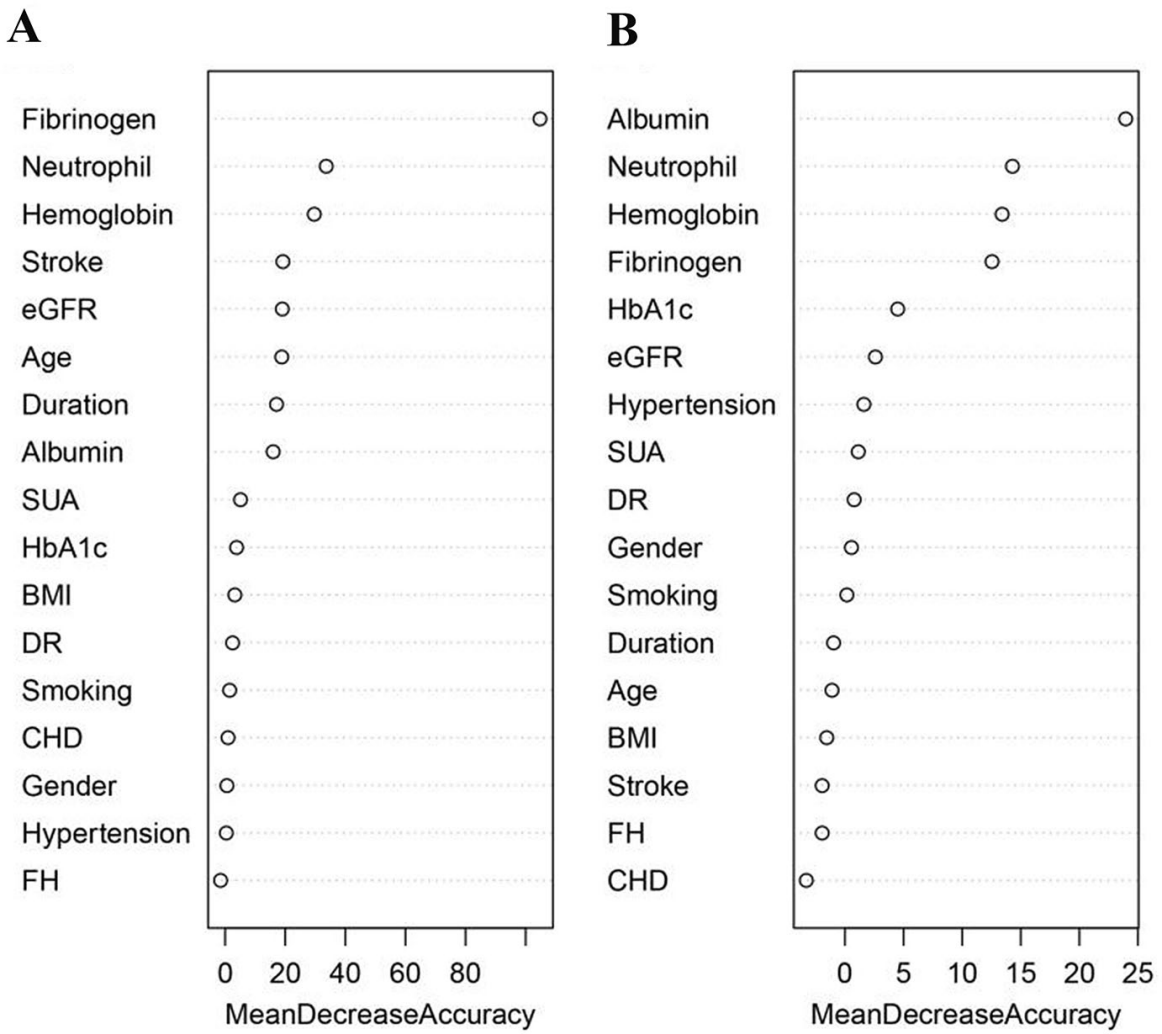
Importance of the variables in assessing the risk and severity of diabetic foot ulcers in the random forest model. **A** Importance measured by mean decrease accuracy for risk of diabetic foot ulcers; **B** Importance measured by mean decrease accuracy for severity of diabetic foot ulcers. *BMI* body mass index, *CHD* coronary heart disease, *DR* diabetic retinopathy, *eGFR* estimated glomerular filtration rate, *FH* family history of diabetes, *HbA1c* hemoglobin A1c, *SUA* serum uric acid

**Fig. 3:**
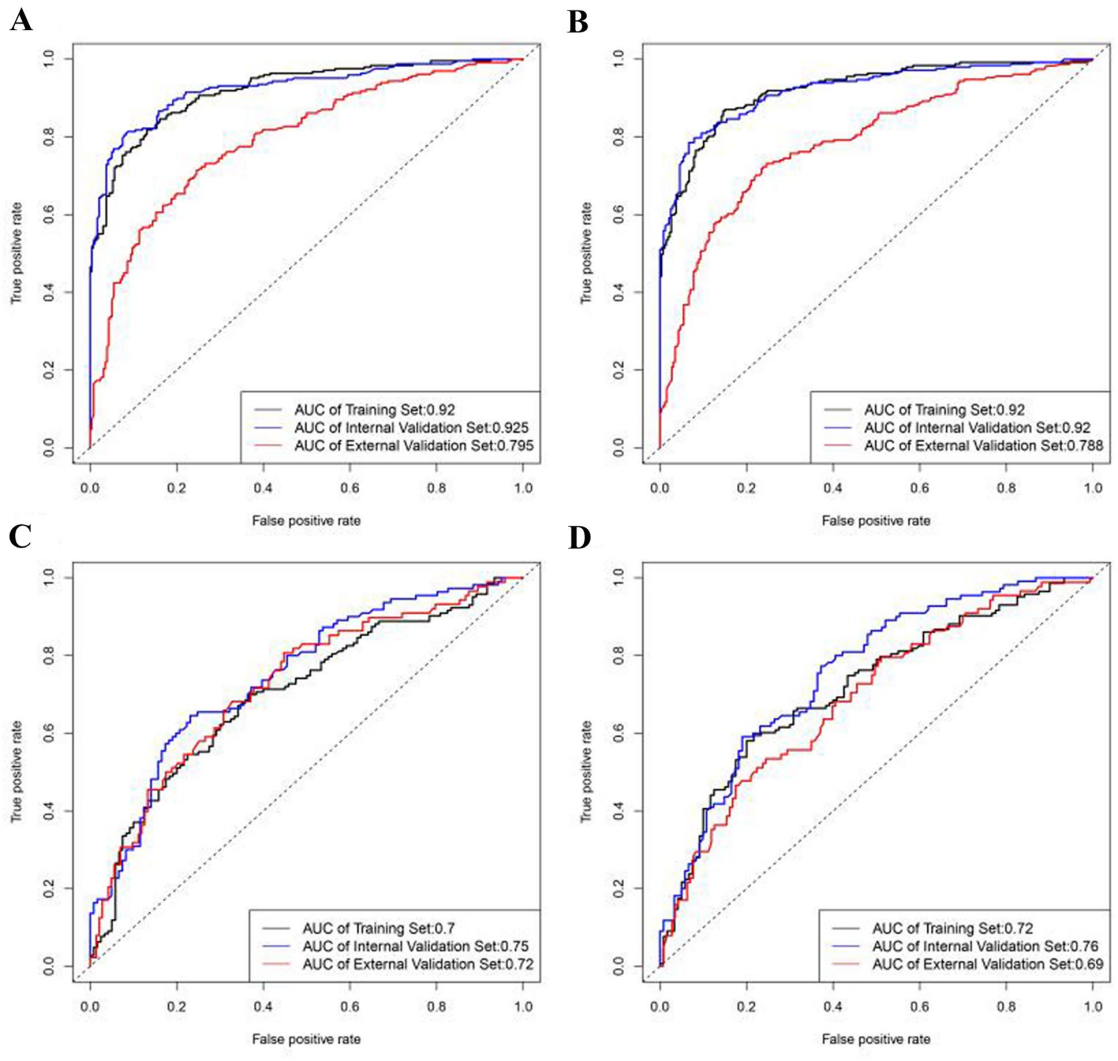
ROC curves for the evaluation of risk and severity of DFUs using random forest model. **A** ROC curves for assessing DFU risk using all 17 variables. **B** ROC curves for assessing DFU risk using eight selected variables. **C** ROC curves for assessing DFU severity using all 17 variables. **D** ROC curves for assessing DFU severity using 10 selected variables. *DFUs* diabetic foot ulcers, *ROC* receiver operating characteristic

We estimated the weights of risk factors related to the occurrence and severity of DFUs, and summarized them in Fig. 2. The top eight risk factors for DFU onset when measured by mean decrease accuracy were plasma fibrinogen level, neutrophil percentage and hemoglobin level in whole blood, stroke, eGFR, age, duration of diabetes, and serum albumin level (Fig. 2A). For DFU risk assessment, the AUC of the internal validation set (AUC = 0.925) was very similar to the training set (AUC = 0.920). However, the AUC decreased substantially in the external validation set (AUC = 0.795, Fig. 3A). This pattern was replicated for the RF model with reduced number of variables, and significant difference could still be identified between the AUC of the external (AUC = 0.788) and internal validation sets (AUC = 0.920) (Fig. 3B). Although the performance of the RF model with full variables (N = 17) was slightly better when compared to the RF model with reduced number of variables (N = 8), the 8-variable RF model was sufficiently effective for determining DFU risk.

Furthermore, the top 10 risk factors for determining DFU severity when measured by mean decrease accuracy were serum albumin level, neutrophil percentage and hemoglobin level in whole blood, plasma fibrinogen level, HbA1c, eGFR, hypertension, SUA level, DR, and sex (Fig. 2B). For evaluating DFU severity, the AUC in the training, internal validation, and external validation sets were very similar in both, the RF model with full variables (N = 17) (Fig. 3C) and the RF model with lesser variables (N = 10) (Fig. 3D), which indicated that the model including 10 risk factors was sufficient to determine DFU severity. The performance of the RF model for evaluating DFU risk was in general better than that for evaluating DFU severity. The complete results of the performance of these RF models are summarized in Additional file 1: Table S2.

### Clinical significance of fibrinogen

**Fig. 4:**
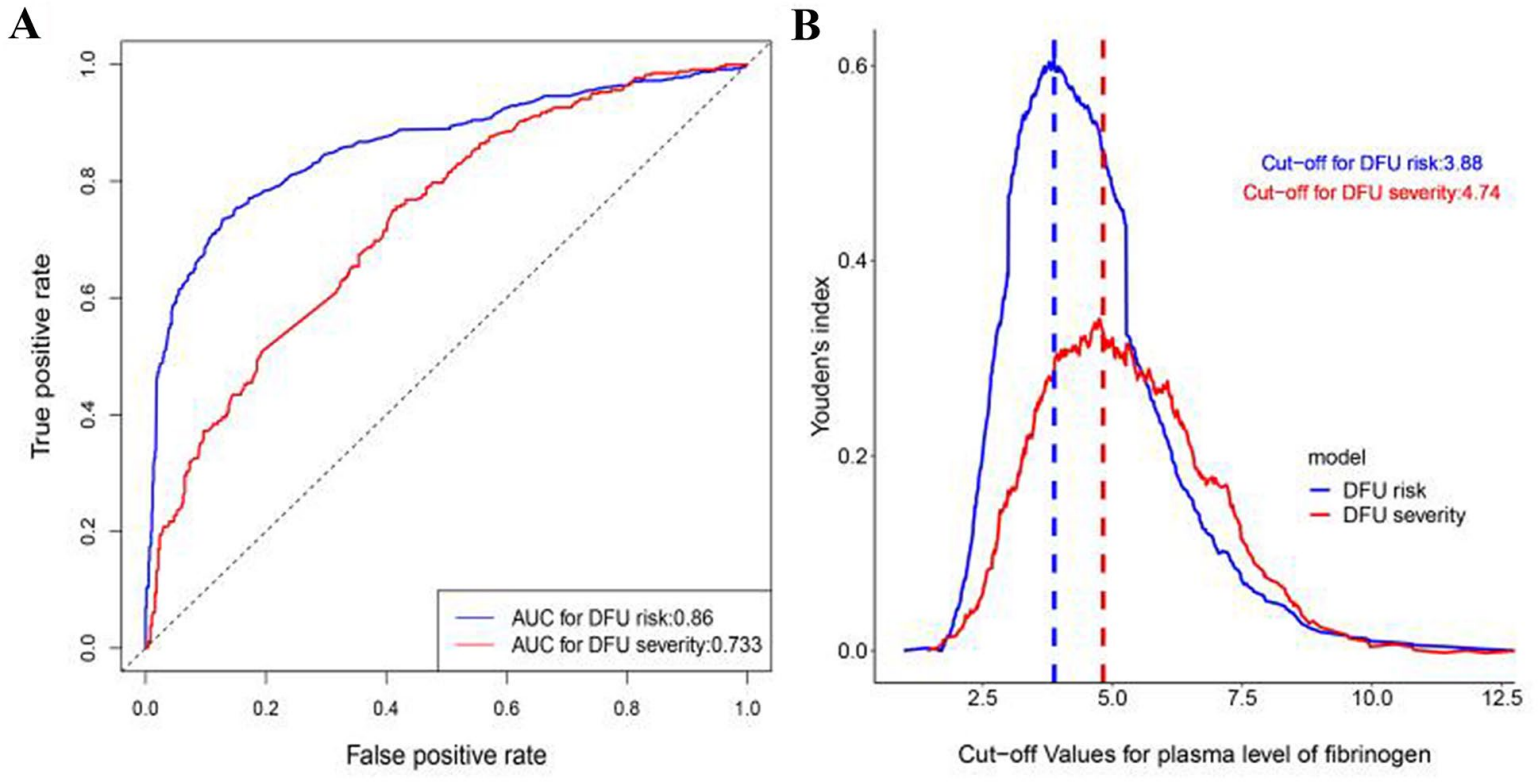
Results of ROC analyses for plasma level of fibrinogen to evaluate DFU risk and severity. **A** ROC curves for plasma level of fibrinogen to evaluate DFU risk and severity using combined data from Set1 and Set2. **B** Youden’s index based on different cut-off values for plasma level of fibrinogen to evaluate DFU risk and severity using combined data from Set1 and Set2. *DFU* diabetic foot ulcer; *ROC* receiver operating characteristic

Since plasma fibrinogen level was found to play a very important role in determining DFU risk and severity in the RF models established by us in this study, we have also evaluated its performance as a single variable model using the combined dataset (N = 1488) of Set1 and Set2. For determining DFU risk, the AUC value using plasma fibrinogen as a single risk factor was 0.86 (95% CI 0.84–0.88) in the model, the sensitivity was 0.74 (95% CI 0.70–0.79), and the specificity was 0.87 (95% CI 0.82–0.90). For determining DFU severity, the AUC value using plasma fibrinogen as a single risk factor was 0.73 (95% CI 0.70–0.77) in the model, the sensitivity was 0.76 (95% CI 0.70–0.79), and the specificity was 0.58 (95% CI 0.46–0.84) (Table 2 and Fig. 4A). Meanwhile, the cut-off values for plasma fibrinogen levels obtained by maximizing Youden’s index were 3.88 g/L and 4.74 g/L for determining DFU risk and severity, respectively (Fig. 4B).

**Table 2:** Overall accuracy and ROC analyses of fibrinogen to determine DFU risk and severity

Model	Cut-off	AUC	Sensitivity	Specificity	ACC
DFU risk	3.88	0.86 [0.84, 0.88]	0.74 [0.70, 0.79]	0.87 [0.82, 0.90]	0.81 [0.79, 0.83]
DFU severity	4.74	0.73 [0.70, 0.77]	0.76 [0.48, 0.88]	0.58 [0.46, 0.84]	0.66 [0.64, 0.70]

*ACC* accuracy, *AUC* area under curve, *DFU* diabetic foot ulcer, *ROC* receiver operating characteristic

## Discussion

This study comprised three major findings. (1) We established a potent weighed risk model to evaluate DFU onset, and the top five risk factors in the model were plasma fibrinogen, neutrophil percentage, hemoglobin, stroke, and eGFR. (2) We also developed an effective weighted risk model to assess DFU severity (Wagner classification ≥ 3), and the top five risk factors in the model were serum albumin, neutrophil percentage, hemoglobin, plasma fibrinogen, and HbA1c. (3) The optimal cut-off values of plasma fibrinogen for determining DFU risk and severity were 3.88 g/L and 4.74 g/L, respectively. Our findings clarified the weighted relationship between risk factors for evaluating DFU risk and severity, which can be used to develop precise strategies for the prevention and treatment of DFUs.

Fibrinogen is known as a procoagulant factor, acute phase response protein and inflammatory factor [17], and an important risk factor of cardiovascular diseases [18]. Diabetes is often accompanied by serious cardiovascular diseases including PAD, which is one of the causes of DFUs [19]. Previous clinical data supported that fibrinogen was a risk factor for diabetic foot disease [20] and acute foot ulcers, and their severity was associated with elevated fibrinogen [21]. Having said that, previous studies did not clearly define the weighted relationship of plasma fibrinogen in the onset and progression of DFUs caused by multiple risk factors, nor did they provide the exact cut-off values to warn diabetic patients about the risk of DFU onset and progression. In this study, we demonstrated that plasma fibrinogen was not only a risk factor for the onset and severity of DFUs, but it also had a high weight in the co-participation of multiple risk factors, especially in the risk model for DFU severity. A possible explanation for this result is that wound infection, inflammatory response, and PAD were more severe in patients with severe DFUs than in patients with mild DFUs. These differences in severity lead to a significant variation in plasma fibrinogen levels, which may guide risk monitoring in DFU patients. Moreover, the present study also calculated the optimal cut-off values of plasma fibrinogen for determining DFU risk and severity using a large sample size. According to the cut-off values, the onset and severity of DFUs can be accurately assessed in clinical practice to achieve early prevention and treatment intervention. However, the latest guidelines for diabetic foot disease do not consider plasma fibrinogen levels in DFU risk stratification and lowering plasma fibrinogen as a treatment option [22], which may be owing to the lack of related high-quality research. Thus, the present study can supplement DFU risk classification and treatment strategies.

This study revealed that low serum albumin was a primary risk factor for DFU severity. Protein deficiency reflected by hypoalbuminemia contributed towards reduced collagen formation and wound dehiscence, and resulted in poor wound healing [23]. Early administration of albumin was seen to enhance wound healing in rats with burn injuries [24]. Hypoproteinemia in DFU patients may be caused by malnutrition or albuminuria caused by diabetic nephropathy. Hypoproteinemia can lead to skin edema, which may further increase the skin’s susceptibility to injury and affect wound healing. Furthermore, hypoproteinemia also increases the risk of the methicillin-resistant *Staphylococcus aureus* infection in DFU patients [25]. Thus, a holistic examination of the patient is important to assess and correct the causes of tissue damage, including nutrition which must be adequate to provide sufficient protein to support the growth of granulation tissue [8]. Taken together, this study demonstrated that patients with severe DFUs have significantly decreased serum albumin, and that improving albumin levels in these patients may benefit wound healing and prevent wound progression and even amputation.

This study was the first to demonstrate, in a large population, that neutrophil percentage was a significant risk factor for the occurrence and progression of DFUs. Since neutrophil percentage represents the presence of infection, the result suggested that DFUs were often associated with infection, especially in severe DFUs. Studies have provided some evidence that increasing severity of infection was associated with higher levels of inflammatory markers [26]. In the current guidelines, the use of white blood cell levels as an indicator to distinguish the severity of DFU infection remains controversial [27], which may be attributed to the small sample size of the relevant studies and whether the study population had applied antibiotics before observation. However, the results of our study indicated that neutrophil percentage was a powerful indicator in determining the occurrence and severity of DFUs.

This study also demonstrated that decreased hemoglobin level ranked third in the risk models for DFU onset and severity. The cause of hemoglobin decline in DFU patients may be due to renal anemia, chronic disease anemia, and blood loss through the wound. Decline in hemoglobin in DFU patients can lead to further hypoxia in ischemic foot tissue, which can result in difficultly in wound healing, and even necrosis or amputation. Previous studies proved that anemia was related to DFU risk and adverse outcomes [28]. A meta-analysis of 2895 patients showed an association between anemia and DFUs, a correlation between the severity of anemia and the severity of DFUs, and that anemia may be a predictor of amputation or death in patients with DFUs [29]. However, whether improvement of anemia can prevent the occurrence and progression of DFUs still needs to be clarified by further prospective, randomized controlled studies.

This study demonstrated that eGFR was also an important risk factor for DFU onset and severity. Some studies supported that there was a strong association between foot ulcers and end-stage renal disease [30, 31]. A prospective study with a 3-year follow-up demonstrated that moderately (30–59) and severely reduced eGFR (< 30) in patients with DFUs were independent predictors for poor prognoses such as healing failure and death [10]. However, the study did not find any association between mildly reduced (60–89) eGFR and poor outcomes in patients with DFUs. A 22-year long prospective study on 1461 male diabetic patients without foot ulcer found that a 1-SD decrease in eGFR increased amputation risk [32]. Diabetic microvascular complications such as diabetic kidney disease and DR were common among DFU patients, and there was a significant association between these complications and diabetic peripheral neuropathy (DPN) and PAD [33], suggesting that diabetic microvascular complications may have a common basis with DPN and PAD, and may be involved in the development and progression of DFUs.

The relationship between blood glucose and DFUs is also an important issue. This study demonstrated that HbA1c was not significant in determining DFU risk, and its weight was lower than that of diabetic duration, a variable that played an indispensable role in the accuracy of the risk model we built. Moreover, the present study also indicated that HbA1c had a higher weight in assessing DFU severity than it did in evaluating DFU risk, which may be due to acute stress response and severe infection in patients with severe DFUs, leading to short-term elevation of blood glucose levels. Increasing evidence showed that intensive glycemic control delayed the onset and progression of diabetic retinopathy, nephropathy, and neuropathy in patients with insulin-dependent diabetes [34, 35], and slow progression of neuropathy in some patients with type 2 diabetes [36]. The benefits of strict glycemic control persisted for a long-term with a reduction in the risk of DPN [37]. These findings suggested that strict glycemic control reduces the risk of diabetic microangiopathy and neuropathy, the diabetes-related complications that underlie the development of DFUs. Although blood glucose control is beneficial to the etiology of diabetic foot disease, the relationship between glycemic control and diabetic foot outcomes remains controversial. Some studies have shown a direct association between baseline HbA1C levels and the rate of wound healing [38], baseline HbA1C levels and amputation rate [39], and mean HbA1C levels and amputation rate [40]. However, other studies found no such association between HbA1C and wound outcomes [41, 42]. The discordance in previous studies indicates that short-term glucose control does not significantly affect DFU outcomes, which may require long-term glucose control and comprehensive risk factor intervention to achieve a good outcome. The present study showed that the combination of multiple risk factors with different weights determined the accuracy of the risk model, supporting that comprehensive risk factor control may reduce DFU risk and severity.

Our study had several strengths. First, the study population was large, which facilitated internal and external validation of the results. Second, we applied superior machine learning algorithms to establish and validate the risk models. Previous studies have indicated that the RF model outperformed conventional regression methods and some similar machine learning algorithm in scenarios of clinical applications [43, 44]. Third, the results of this study such as fibrinogen and neutrophil percentage complement the guidelines for DFU risk stratification. Lastly, our study provided optimal cut-off values of plasma fibrinogen for determining DFU risk and severity, which may be groundbreaking evidence for further studies that may, in due time, lead to updating of the guidelines.

There were some limitations in this study. One major limitation of the present study is that cross sectional data were utilized, and therefore this study does not allow setup the time sequence between selected risk factors and DFU occurrence. In future prospective studies are required to establish this link. Second, we did not obtain information on lower extremity angiography and electromyography of the participants, long-term blood glucose control indicators such as the time of blood glucose normalization, and medication information, which may have reduced our ability to explore other risk factors. Third, this study was a retrospective study, therefore no cause-and-effect relationship could be established. Lastly, all the participants in this study were from China, and there may be genetic and environmental differences among diabetic patients in other countries. Therefore, the results of this study need to be verified in other countries and ethnic groups.

## Conclusions

In summary, we have developed potent weighted risk models for assessing the occurrence and severity of DFUs. These findings provided important evidence for prioritizing strategies for the prevention and treatment of DFUs, which may help reduce poor outcomes in diabetic patients. Further studies are needed to evaluate whether intervention with important risk factors can reduce DFU onset and progression.

## Supplementary Information

**Additional file 1. **Detail information on parameter selection and optimization, variable selection and model performance measured by multiple indicators

## Data Availability

The datasets used during the current study are available from the corresponding author on reasonable request.

## Abbreviations

AUC: Area under curve
BMI: Body mass index
CI: Confidence interval
DFU: Diabetic foot ulcer
DPN: Diabetic peripheral neuropathy
DR: Diabetic retinopathy
eGFR: Estimated glomerular filtration rate
HbA1c: Hemoglobin A1c
NPDR: Non-proliferative diabetic retinopathy
PDR: Proliferative diabetic retinopathy
PAD: Peripheral arterial disease
RF: Random forest
SD: Standard deviation
SUA: Serum uric acid
