# Supplementary material for: A potent weighted risk model for evaluating the occurrence and severity of diabetic foot ulcers
Source: Diabetol Metab Syndr. 2021 Aug 31;13:92. doi: 10.1186/s13098-021-00711-x (PMC8407043; doi:10.1186/s13098-021-00711-x)
Supplement: Supplementary file 1 — Additional file 1. Detail information on parameter selection and optimization, variable selection and model performance measured by multiple indicators [file 13098_2021_711_MOESM1_ESM.docx]

**Additional Methods**

**Parameter Selection and Optimization**

In general, two parameters of RF models were needed to be set. The first was the parameter "mtry". This parameter indicated the number of variables randomly sampled as candidates at each split. In the present study, we tuned our "mtry" value by trying different values and compared the model performance. The results are indicated in Additional Figure S1 (for evaluation of DFU risk) and S2 (for evaluation of DFU severity). As we can see from the two plots, the minimized mean error rates of the RF models were obtained at mtry=6 and 8 for the evaluation of DFU risk and severity, respectively. Therefore, we chose two values for our RF model.

Another parameter was "ntree", which indicated the number of trees to grow. As we know, because the output of an RF model is based on the average value of each tree, more trees indicate more stable results. For the present study, we utilized the default value of 1000 for "ntree".

**Variable Selection for RF Models with Reduced Number of Variables**

Although an RF model was considered to be insensitive to overfitting, we still constructed RF models with a reduced number of variables because of the difference in performance of our full RF model in the internal and external validation set. The selection of variables in the RF models was based on the importance of each variable in the full RF model (Figure 2) and the average model prediction errors when variables were removed from the RF models. For the evaluation of DFU risk, when we kept eight or more variables in the RF model, the prediction error did not change significantly (Additional Figure S3). Thus, for the RF model with a reduced number of variables, we selected eight variables according to their importance in the complete RF model. For the evaluation of DFU severity, when we kept 10 or more variables in the RF model, the prediction error did not change significantly (Additional Figure S4). Thus, for the RF model with a reduced number of variables, we selected 10 variables according to their importance in the full RF model.

**Additional Table S1.** Relevant information of the 17 variables that was utilized in the random forest model.

| No. | Variables | Description | Type | Values |
| --- | --- | --- | --- | --- |
| 1 | Sex | Sex | Categorical | 0:female,1:Male |
| 2 | Age | Age in years | Continuous | - |
| 3 | BMI, kg/m^2^ | Body mass index | Continuous | - |
| 4 | Diabetes duration, year | Time since diagnosis | Continuous | - |
| 5 | Coronary heart disease | Coronary heart disease | Categorical | 0:No,1:Yes |
| 6 | Stroke | Stroke | Categorical | 0:No,1:Yes |
| 7 | Hypertension | Hypertension | Categorical | 0:No,1:Yes |
| 8 | Diabetic retinopathy | Diabetic retinopathy | Categorical | 0:No,1:Yes |
| 9 | Family history of diabetes | Family history of diabetes | Categorical | 0:No,1:Yes |
| 10 | Smoking status | Smoking status | Categorical | 0:No,1:Yes |
| 11 | Hemoglobin, g/L | Hemoglobin level in whole blood | Continuous | - |
| 12 | Neutrophil percentage | Percentage of neutrophil in whole blood | Continuous | - |
| 13 | Serum albumin, g/L | Serum level of albumin | Continuous | - |
| 14 | Serum uric acid, μmol/L | Serum level of uric acid | Continuous | - |
| 15 | Plasma fibrinogen, g/L | Plasma level of fibrinogen | Continuous | - |
| 16 | eGFR, mL/min/1.73m^2^ | Estimated glomerular filtration rate | Continuous | - |
| 17 | HbA1c, % | Percentage of glycated hemoglobin in whole blood | Continuous | - |

BMI: Body mass index; eGFR: Estimated glomerular filtration rate; HbA1c: Hemoglobin A1c.

Additional Table S2. Performance of the models measured by multiple indicators.

| Models | Dataset | AUC | Youden's index | Sensitivity | Specificity | NPV | PPV | Accuracy |
| --- | --- | --- | --- | --- | --- | --- | --- | --- |
| Full model for risk | Training Set | 0.92[0.89-0.94] | 0.69[0.64-0.76] | 0.82[0.73-0.91] | 0.87[0.79-0.95] | 0.84[0.78-0.91] | 0.85[0.79-0.94] | 0.84[0.82-0.88] |
|  | Internal Validation Set | 0.92[0.90-0.95] | 0.73[0.68-0.79] | 0.80[0.75-0.92] | 0.92[0.80-0.97] | 0.82[0.78-0.92] | 0.92[0.81-0.96] | 0.86[0.84-0.90] |
|  | External Validation Set | 0.79[0.76-0.83] | 0.47[0.42-0.56] | 0.71[0.55-0.81] | 0.75[0.67-0.90] | 0.75[0.67-0.82] | 0.72[0.66-0.85] | 0.74[0.71-0.78] |
| 8 features model for risk | Training Set | 0.92[0.90-0.94] | 0.72[0.66-0.78] | 0.87[0.79-0.91] | 0.85[0.81-0.92] | 0.88[0.82-0.92] | 0.84[0.80-0.91] | 0.86[0.83-0.89] |
|  | Internal Validation Set | 0.92[0.90-0.94] | 0.72[0.67-0.78] | 0.79[0.75-0.88] | 0.93[0.84-0.96] | 0.81[0.78-0.88] | 0.92[0.84-0.95] | 0.86[0.83-0.89] |
|  | External Validation Set | 0.79[0.75-0.83] | 0.49[0.43-0.57] | 0.73[0.58-0.79] | 0.75[0.72-0.89] | 0.76[0.69-0.81] | 0.73[0.69-0.84] | 0.74[0.72-0.79] |
| Full model for severity | Training Set | 0.70[0.63-0.77] | 0.33[0.26-0.47] | 0.70[0.41-0.79] | 0.63[0.58-0.91] | 0.64[0.54-0.73] | 0.69[0.64-0.86] | 0.67[0.61-0.73] |
|  | Internal Validation Set | 0.75[0.68-0.81] | 0.41[0.33-0.54] | 0.65[0.54-0.89] | 0.77[0.50-0.88] | 0.70[0.63-0.85] | 0.72[0.60-0.83] | 0.71[0.66-0.78] |
|  | External Validation Set | 0.72[0.65-0.79] | 0.36[0.28-0.50] | 0.81[0.47-0.90] | 0.55[0.49-0.88] | 0.82[0.70-0.90] | 0.53[0.46-0.73] | 0.65[0.61-0.76] |
| 10 features model for severity | Training Set | 0.72[0.66-0.78] | 0.38[0.29-0.50] | 0.58[0.46-0.79] | 0.80[0.58-0.90] | 0.62[0.55-0.72] | 0.78[0.67-0.87] | 0.68[0.63-0.74] |
|  | Internal Validation Set | 0.76[0.69-0.82] | 0.40[0.33-0.54] | 0.59[0.55-0.92] | 0.81[0.48-0.87] | 0.69[0.64-0.89] | 0.74[0.58-0.81] | 0.71[0.66-0.77] |
|  | External Validation Set | 0.69[0.62-0.76] | 0.29[0.22-0.44] | 0.47[0.43-0.88] | 0.83[0.43-0.89] | 0.72[0.67-0.89] | 0.62[0.44-0.73] | 0.69[0.58-0.74] |

AUC: area under curve; NPV: negative predictive value; PPV: positive predictive value.

Youden's index, sensitivity, specificity, NPV, PPV, and accuracy were reported and calculated at the maximum value of Youden's index.

All the indicators were presented with its 95% confidence interval.


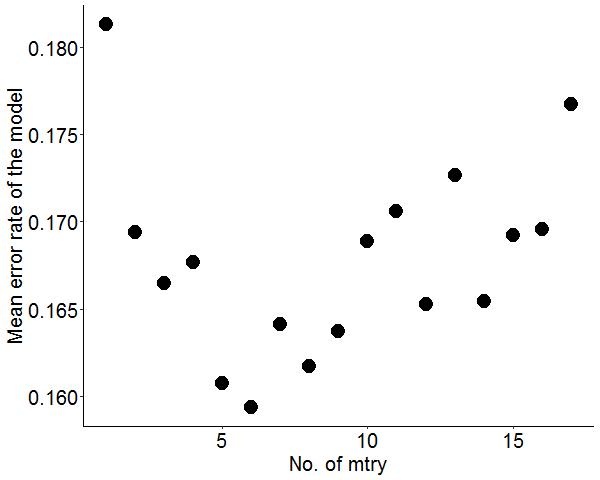


**Additional Figure S1.** Model parameter optimization: Selection of the number of mtry to determine the risk of diabetic foot ulcers


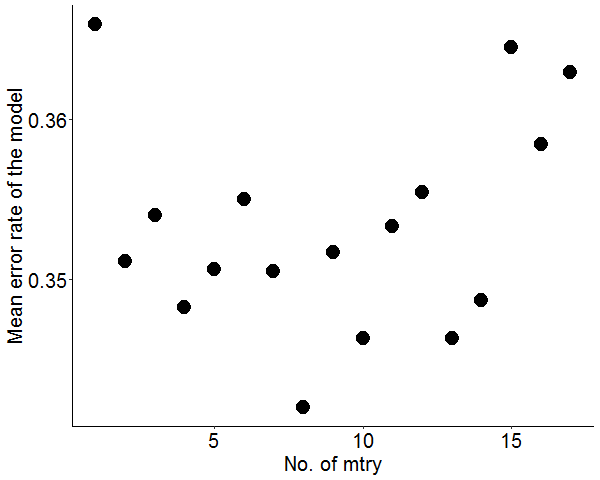


**Additional Figure S2.** Model parameter optimization: Selection of the number of mtry to determine the severity of diabetic foot ulcers


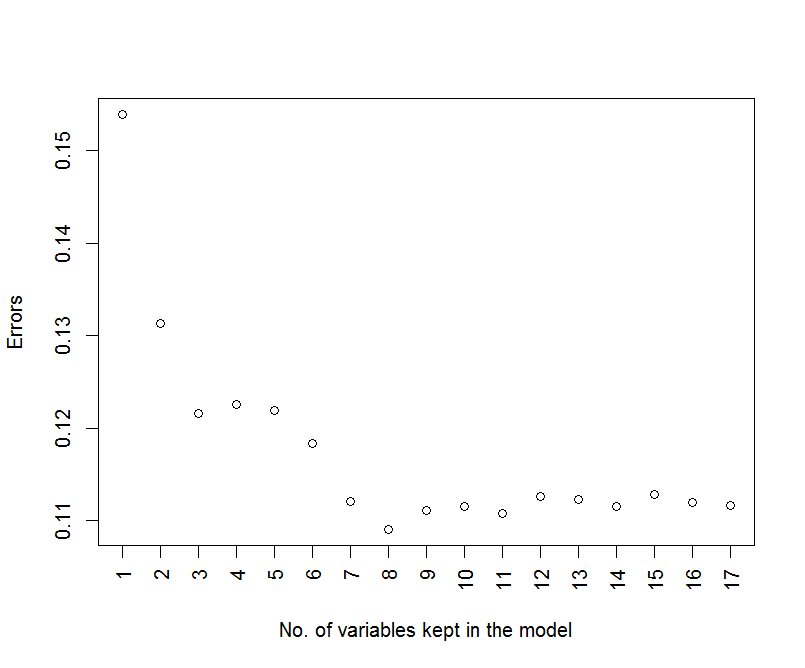


**Additional Figure S3.** Variable selection: Number of variables kept in the model versus average model errors in evaluating diabetic foot ulcer risk.

.


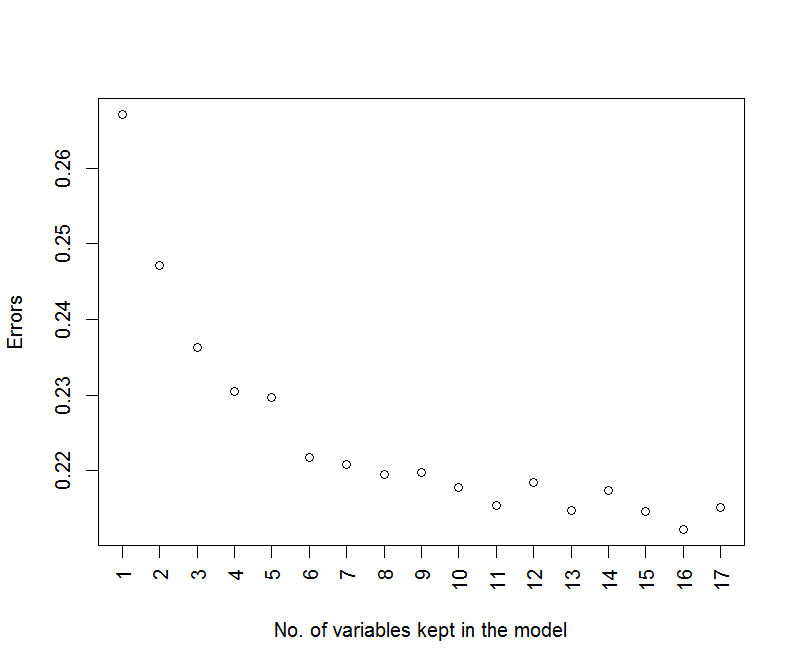


**Additional Figure S4**. Variable selection: Number of variables kept in the model versus average model errors in evaluating diabetic foot ulcer severity.
